# Middle ear implants: functional gain in mixed hearing loss

**DOI:** 10.1590/S1808-86942012000100017

**Published:** 2015-10-20

**Authors:** Mario Emilio Zernotti, Maria Fernanda Di Gregorio, Andrea C. Bravo Sarasty

**Affiliations:** a Doctor and Professor (Professor of Otorhinolaryngology. Catholic University of Cordoba. Argentina); b Medico (Staff ENT Department Sanatorio Allende); c Fonoaudiologist (Especialist in Audiology)

**Keywords:** hearing loss, mixed conductive-sensorineural, otitis media, prostheses and implants, suppurative

## Abstract

Osseous atresia and chronic otitis media are diseases benefit with middle ear implants. Surgery for atresia is technically complicated, has significant number of complications and functional results are often poor. The osseointegrated hearing aids are an alternative. They provide a very good functional gain, but have many problems with the skin and osseointegration. In chronic otitis media, the ossiculoplasty solved partially the hearing problem. Unfortunately in some cases of otitis media and in open cavities fitted with conventional hearing aids the gain is unsatisfactory.

**Aim:**

To determine the usefulness of an active middle ear implant.

**Material and method:**

Longitudinal Study. Vibrant-Soundbrigde was implanted in 8 patients with severe mixed hearing loss. 4 patients had chronic otitis media and 4 had unilateral atresia. The placement of the stimulator (FMT or Floating Mass Transducer) was in 5 patients on round window, 2 in stapes and one in the oval window.

**Results:**

Functional gain was 35 dB, 40 dB, 48.7 dB and 50 dB for the frequencies 500, 1000, 2000 and 4000 Hz, respectively.

**Conclusion:**

Vibrant-Soundbrigde is an excellent option in hearing recovery in severe and profound mixed hearing loss. It also provides an excellent functional gain in diseases difficult to treat with conventional hearing aids.

## INTRODUCTION

With the advent of middle ear implants (MEI) many diseases have been benefited from these devices. Osseous atresia and the wide chapter of chronic otitis media are the principal groups of diseases benefit from this type of hearing aid. Every day the indications are increasing and with it the experience of hearing restoration in different pathologies. Although that in the external atresia we have a wide array of treatment options, they have not always been satisfactory especially in terms of functional outcomes. Thus we have that the surgery for atresia is technically complicated, it also has a significant rate of complications and functional results finally are often poor. The osteointegrated hearing aids (BAHA and Ponto) are an alternative to conventional surgery.

These devices provide a very good functional gain and are easy to install, but has a high rate of skin problems and difficulties with osteointegration. Finally, many patients rejected this kind of devices for aesthetic reasons.

On the other hand, in chronic otitis media, exist and have been described numerous techniques and prostheses to restore hearing. The ossiculoplasty with many variants of prosthesis (TORP, PORP) and different materials (hydroxyapatite, titanium, Teflon, steel, etc.) have only partially solved the problem. While we have established and standardized techniques, functional failure often leads the patient to disappointment as the only alternative being the conventional hearing aid. Unfortunately in some of these otitis media, especially those who are sequelae at tympanic membrane (perforations or tympanosclerosis) or large open cavities, the use of conventional hearing aids is difficult and often unsatisfactory.

For all these reasons is that they have appeared and developed a group of middle ear implantable hearing aids. The common characteristic is that the amplification mechanism must be housed in the middle ear. This prosthesis uses different mechanisms such as mechanical, electromagnetic, and piezoelectric stimulation. In this paper we have used the device Vibrant-Soundbrigde (VSB) which uses vibratory energy to stimulate the inner ear and allows a high possibility of implantation sites at the middle ear. The Floating Mass Transducer (FMT) could be placed in ossicular chain, in round or oval window or joint to any middle ear prosthesis. The study aims to determine the utility through the functional gain in patients implanted with VSB with severe mixed hearing loss, where conventional hearing aids cannot be used or do not provide adequate results.

## MATERIAL AND METHODS

We use Vibrant-Soundbrigde device, manufactured by Med-el, Austria. This device is an implantable hearing aid consists of two main parts. An external processor with the battery, the electronic and the microphone, called AP 404 or Amadé, and an implantable part which consists of two key components the VORP (Vibrating Ossicular Replacement Prosthesis) and FMT (Floating Mass Transducer).

The VORP is connected to a cable at the distal end with the floating mass transducer (FMT). This is the electromagnetic coil can be placed on any mobile portion of the middle ear, ie the ossicles or windows, or you can join the FMT to any ossicular replacement prosthesis available.

Initial results are presented in 8 patients implanted with the VSB with severe mixed hearing loss. The age of the patients was from 12 to 57, with an average of 41.3 years. We have operated five men and three women. Four of these patients had chronic otitis media with cholesteatoma, while the other four suffered from unilateral atresia of external auditory canal.

All patients suffering from chronic otitis media have been previously conventional hearing aid users with poor results as well as active (wet) cavities during use. In these cases the VSB was putting after removed of cholesteatoma in a second stage. The main reason is the necessity of dry cavities to avoid explantations. Finally, in the open cavities is essential to make a bony groove to place the cable, and then cover it with bone pate a cartilage.

Patients with unilateral atresia had not used any type of device to improve hearing.

The placement of the stimulator (FMT) was in 5 patients in the round window, 2 in stapes and the remaining in the oval window.

The technique in patients with the floating mass transducer (FMT) in the round window consists of some very important steps. The most important step, is the exposure of the round window membrane generously, because one end of the FMT should be fully contacted on the surface of membrane to conduct properly the energy to the inner ear. The other step is to protect the membrane with a piece of temporalis fascia to prevent damage. In this manner it has great physical link with very good sound conduction.

In the technique on the stapes, we put the FMT directly attached by titanium clip to some crura or on the capitel of the stapes. It is necessary to note that this alternative is common when there is no ossicular chain, usual in radical mastoid surgery.

Finally in one patient we had to place the prosthesis on the footplate (not a visible round window). We carry out a total stapedectomy and covered with fascia the oval window, on which we place the FMT.

## RESULTS

Audiometric thresholds were measured pre-and post-implant in the frequencies 500, 1000, 2000 and 4000 Hz. Postoperative measurements were performed at 30, 60 and 90 days, settling for this work measurements of the third month.

**Figure 1:**
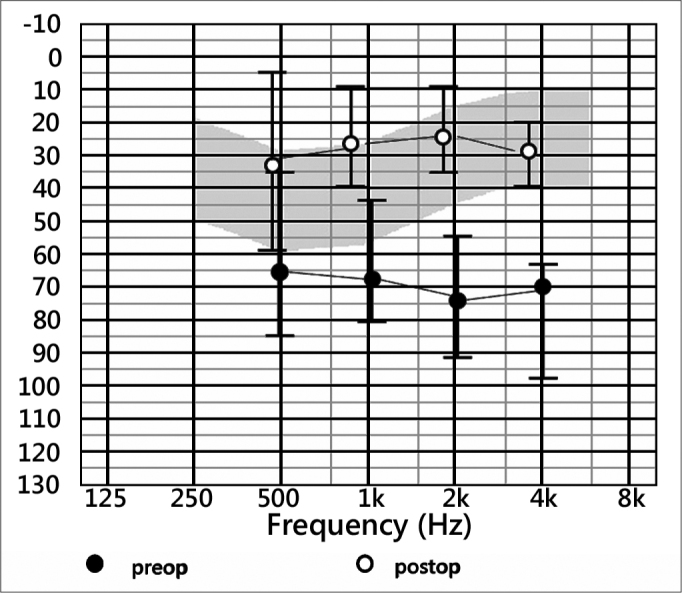
Pre and Postoperative thresholds - average thresholds in preoperative and postoperative audiograms.

**Figure 2:**
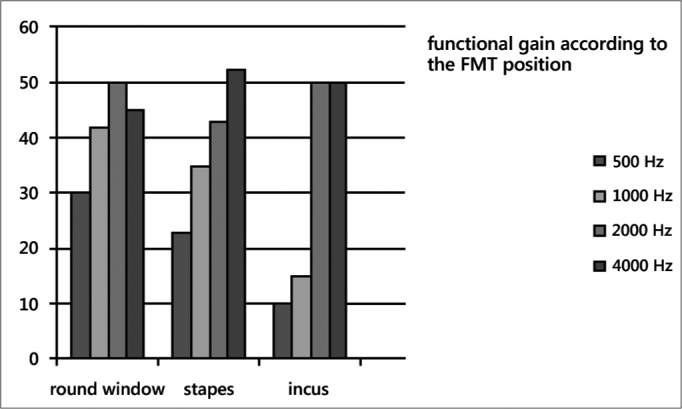
functional gain - Functional gain according to the different placements of the prosthesis.

With regard to pre-surgical measurements should be mentioned that in only one patient had an extra hearing loss of 10 dB in 2000 Hz and 15 dB at 4000 Hz attributable to the surgical maneuver and corresponded to a patient with FMT on stapes placement. The remaining patients remained unchanged from their pre-surgical thresholds. These preoperatively thresholds were on average in 8 patients of 67.5 dB, 68.1 dB,73.1 dB and 80 dB respectively. In the postoperative measurements average threshold were 32.5 dB at 500 Hz, 28.1 dB at 1000 Hz, 24.3 dB at 2000 Hz and 30 dB in 4000 Hz. (Figure 1). So functional gain was 35 dB, 40 dB, 48.7 dB and 50 dB for the frequencies 500, 1000, 2000 and 4000 Hz, respectively. Regarding to the site of implantation the FMT the changes were small, with better performance placing on the round window, where on average earned 30 dB, 42 dB, 50 dB and 45 dB at 500, 1000, 2000 and 4000 Hz (Figure 2).

## DISCUSSION

With regard to surgery in atresia, there is a long way, unfortunately not always successful. Siegert^1^, in a recent work proposes three surgical steps, including treatment of microtia in plastic way. In this publication, the author reports that 76% of patients get a hearing with thresholds near 30 dB.

In a review conducted at Oklahoma University, only 50% of patients reaches threshold near 30 dB^2^.

Yellow et al published results in atresioplasty surgery with pure tone average thresholds and bone-air gap of 37.5 and 29.4 dB, respectively^3^.

Regarding to osseointegrated hearing aids, should be mentioned that there are reports claiming that the closure of the gap has reached 85.1% of patients with external atresia^4^.

As seen on functional outcome is good, but this surgery has an important number of major complications. Hobson et al.^5^ mentioned in a series of over 600 implants a complication rate of 23.9%, having a surgical revision rate of 12.1%. Another review mentioned postoperative complications include severe skin infections in 8%, while the problems of osseointegration achieved according to the authors were 18%, plus 8% of patients where the skin covers the external connector^6^.

Finally, it should be mentioned that the support (Abutment) is percutaneous and many patients reject the idea for aesthetic reasons and maintenance. With respect to the use of VSB in atresia in adults and adolescents, Kiefer described 14 patients with post-implant audiometric thresholds below 30dB.^7^. As another recent publication of Frenzel et al., on 7 patients obtained a functional gain of 45.5dB^8^. The same author also published a case of a 6-year with normal thresholds after implantation of the VSB^9^.

Chronic otitis media is generally associated with some degree of hearing loss. Da Costa^10^ said that while the conductive hearing loss can be improved through surgery, sensorineural hearing loss is a permanent defect only improve with conventional hearing aids. There are recent publications with good results with ossicular prostheses. Iñiguez-Cuadra publishes a series of 94 patients where used total ossicular replacement prosthesis of titanium (TORP) in chronic otitis media getting a bone-air gap less than 20dB in 66% of cases^11^.

Alaani et al.^12^ published a study on 97 patients operated with titanium PORP and TORP. The results show an average gap of 10.6dB after surgery for patients with PORP, while the gap in postoperative patients with TORP was higher in the study, reaching 14.84dB. Therefore we can say that this type of prosthesis is very useful to decrease bone-air gap, however we also know that many patients with chronic otitis media suffer from an additional sensorineural hearing loss, which should, after surgery, be equipped with a conventional hearing aid. In these patients the VSB can be very useful.

Streitberger et al.^13^ present a series of patients implanted with VSB suffering chronic otitis media with cholesteatoma. The preoperative thresholds were 82.38dB SPL, thresholds in word recognition were 94.28 dB SPL. This group obtained three months later, audiometric thresholds of 50.63 dB SPL with vocal audio to 61.68 dB. After 6 to 9 months tonal audiometric threshold was 47.89 dB SPL and the word recognition test of 53.33 dB SPL. In our study we obtained no statistically significant differences between implant patients suffering from external auditory canal atresia with those with chronic otitis media cholesteatoma, so the average functional gain in both groups was 35dB, 40dB, 48.7dB and 45dB for the frequencies 500, 1000, 2000 and 4000 Hz, respectively, according to the literature referred here

## CONCLUSION

VSB device is a very important option in hearing recovery in severe and profound mixed hearing loss. It also provides an excellent functional gain in diseases difficult to treat with conventional equipment such as wet radical cavities impossible to equip with molds (in cavities without inflammatory activity) or in the case of external atresia without the possibility of using hearing aids for air.
